# Predictors of five-repetition sit-to-stand test performance in patients with lumbar degenerative disease

**DOI:** 10.1007/s00701-022-05441-1

**Published:** 2022-12-07

**Authors:** Anita M. Klukowska, Victor E. Staartjes, W. Peter Vandertop, Marc L. Schröder

**Affiliations:** 1 Department of Neurosurgery, Bergman Clinics, Amsterdam, Netherlands; 2 Neurosurgery, Amsterdam Movement Sciences, Amsterdam UMC, Vrije Universiteit Amsterdam, Amsterdam, Netherlands; 3 Department of Neurosurgery, Clinical Neuroscience Center, University Hospital Zurich, University of Zurich, Zurich, Switzerland

**Keywords:** Sit-to-stand, Objective test, Degenerative disc disease, Lumbar stenosis, Lumbar disc herniation, Functional impairment

## Abstract

**Background:**

The five-repetition sit-to-stand test (5R-STS) has recently been validated as an objective measure of functional impairment in patients with lumbar degenerative disease (LDD). Knowledge of factors influencing 5R-STS performance is useful to correct for confounders, create personalized adjusted test times, and potentially identify prognostic subgroups. We evaluate factors predicting the 5R-STS performance in patients with LDD.

**Methods:**

Patients with LDD requiring surgery were included. Each participant performed the 5R-STS and completed a questionnaire that included their age, gender, weight, height, body mass index (BMI), smoking status, education level, employment type, ability to work, analgesic drug usage, history of previous spinal surgery, and EQ5D depression and anxiety domain. Surgical indication and index level of the spinal pathology were also recorded. Predictors of 5R-STS were identified through multivariable linear regression.

**Results:**

The cohort consisted of 240 patients, 47.9% being female (mean age, 47.7 ± 13.6 years). In the final multivariable model incorporating confounders, height (regression coefficient (RC), 0.08; 95% confidence interval (CI), 0.003/0.16, *p* = 0.042) and being an active smoker (RC, 2.44; 95%CI, 0.56/4.32, *p* = 0.012) were significant predictors of worse 5R-STS performance. Full ability to work (RC, − 2.39; 95%CI, − 4.39/ − 0.39, *p* = 0.020) was associated with a better 5R-STS performance. Age, height, surgical indication, index level of pathology, history of previous spine surgery, history of pain, analgesic drug use, employment type, and severity of anxiety and depression symptoms demonstrated confounding effect on the 5R-STS time.

**Conclusions:**

Greater height, being an active smoker, and inability to work are significant predictors of worse 5R-STS performance in patients with LDD.

**Trial registration:**

ClinicalTrials.gov Identifier: NCT03303300 and NCT03321357.

**Supplementary information:**

The online version contains supplementary material available at 10.1007/s00701-022-05441-1.

## Introduction

One of the many advancements of spinal surgery in the recent decades is the introduction of objective tests of functional impairment during assessment of patients with lumbar degenerative diseases (LDD), such as the timed-up-and-go test (TUG) and 6-min-walking test (6MWT) [28]. They are simple and straightforward and can account for symptoms such as foot drop or limping that cannot be detected by patient-reported outcome measures (PROMs) but are equally important during decision-making process regarding surgical intervention in LDD [9, 12, 25, 29].

One of the most well-validated tests of objective functional impairment (OFI) for patients with LDD is the five-repetition sit-to-stand test (5R-STS) [27]. It cannot only be used in the initial assessment of OFI in LDD but similarly to monitor recovery and progress after a surgical intervention, which the patients can perform reliably by themselves at home [25]. Given the wide applicability of 5R-STS, it is crucial to understand its prognostic factors in patients with LDD.

First, this would aid in creating person-adjusted test times. An otherwise healthy, adipose 82-year-old cannot be expected to perform the 5R-STS like a healthy athletic 21-year-old. Knowledge of the effect sizes of sociodemographic variables may thus help in the development of expected person-adjusted test times similarly to age-adjusted D-dimer level testing [7, 23]. Second, the significance of some prognostic factors such as employment type or education of 5R-STS in patients with LDD is yet to be analyzed. As over recent years tests of OFI are increasingly being used as outcome measures in clinical trials of patients with LDD, identified prognostic factors can also be used to correct for confounding [28]. The purpose of this study was therefore to evaluate prognostic factors of 5R-STS performance in patients with LDD.

## Materials and methods

### Study design and oversight

In two prospective studies, carried out between October and December of 2017 and between December 2017 and June 2018, patients were seen at a Dutch specialized short-stay outpatient spine surgery clinic [25, 27]. Participants filled in questionnaires right after performing the test containing baseline sociodemographic data: age, gender, BMI, height, weight, smoking status, education level, employment type, ability to work, and EuroQOL-5D (EQ-5D) questionnaire. Pathology and spinal level involvement were also recorded. The prospective cohort studies (ClinicalTrials.gov Identifier: NCT03303300 and NCT03321357) were approved by the local institutional review board (Medical Research Ethics Committees United, Registration Number: W17.107 and W17.134) and were conducted according to the Declaration of Helsinki. Informed consent was obtained from all participants.

### Study population

All enrolled patients were candidates for surgery and were assessed during outpatient consultations. Inclusion criteria were the presence of lumbar disc herniation (LDH), lumbar stenosis, lumbar spondylolisthesis, degenerative disc disease (DDD), or synovial facet cysts, requiring surgical treatment. Patients with hip or knee prosthetics and those requiring walking aides were excluded to eliminate these confounders.

### Testing protocol

The test was performed according to the protocol described by Jones et al. [10, 27]. The participants were asked to sit down on an armless chair of standard height (48 cm) and with a hard seat, firmly placed against a wall. The participants were instructed to fold their arms across their chest and to keep their feet flat on the ground. Participants were required to wear stable shoes for the test. To familiarize with the movement, the participants were asked to stand up fully and sit back down again once without using their upper limbs. If assistance was required or if the maneuver could not be completed, the test was abandoned. Otherwise, the patients were asked to, starting on the command “go,” stand up fully and sit down again, landing on the seat firmly, five times as fast as possible. Using a stopwatch, the five repetitions were timed from the initial command to the completed fifth stand. This time was recorded as the participant’s score. If the patient was unable to perform the test in 30 s, or not at all, this was noted down, and the test score was recorded as 30 s.

### Statistical analysis

Continuous variables are reported as mean ± standard deviation and categorical variables as numbers and percentages. The two studies were pooled to form one cohort. To identify univariable predictors of 5R-STS performance in individuals with LDD, univariable linear regression models were fitted for each of the baseline variables, with 5R-STS time as the dependent variable. Subsequently, multivariable linear regression models were fitted to identify factors independently associated with 5R-STS performance and OFI, based on the purposeful variable selection procedure described by Hosmer and Lemeshow [5]. In more detail, variables were considered for inclusion at univariable *p* ≤ 0.25. Subsequently, a multivariable model was built, and variables that did not have a significant effect (defined as *p* ≤ 0.1) or that did not demonstrate confounding (defined using the change-in-estimate criterion of 20% or greater) were iteratively removed from the model. Finally, any variable not eligible for the original multivariable model was added iteratively, and the model was subsequently reduced in the same way as described above by iterative removal of only those variables that were additionally added [5]. All analyses were carried out using R version 3.6.2 (the R Foundation for Statistical Computing, Vienna, Austria) [22]. A 2-tailed *p* ≤ 0.05 was considered significant. The statistical code is provided (Supplementary Content 1).

## Results

### Cohort

In total, 240 adult patients (47.9% female) with LDD were included in this study. The mean age was 47.7 ± 13.6 years, and the mean BMI was 25.4 ± 3.2 kg/m^2^. Detailed baseline characteristics are provided in Table 1. The overall mean 5R-STS time was 13.04 ± 6.10 s 1.

**Table 1:** Basic demographic data for 240 patients with lumbar degenerative disease. Continuous variables are presented as mean ± SD and categorical variables as frequency (%)

Characteristics	All participants (*n* = 240)	Female (*n* = 115)	Male (*n* = 125)
Age	47.7 ± 13.6	47.8 ± 13.0	47.6 ± 14.2
Gender			
Female	115 (47.9)	-	-
Male	125(52.1)	-	-
BMI (kg/m^2^)	25.4 ± 3.2	25.4 ± 3.4	25.33 ± 3.05
Height (cm)	176.2 ± 10.2	169.2 ± 7.4	182.7 ± 8.0
Weight (kg)	78.9 ± 12.9	72.8 ± 11.0	84.6 ± 12.0
Smoking status			
Active smoker	74(30.8)	33(28.7)	41(32.8)
Ceased smoking	73(30.4)	37(31.2)	36(28.8)
Never smoked	93(38.8)	45(40.1)	48(38.4)
Ability to work			
Full	55(23.0)	25(21.7)	30(24.)
Limited	58(24.1)	25(21.7)	33(26.4)
** Unable**	**127(52.9)**	**65(56.6)**	**62(49.6)**
Indication			
** Disc herniation**	**174 (72.5)**	**80(69.6)**	**94(75.2)**
Stenosis	42(17.5)	24(20.8)	18(14.4)
DDD	13(5.4)	7(6.1)	6(4.8)
Spondylolisthesis	11(4.6)	4(3.5)	7(5.6)
Index level			
L2–3	7(3.0)	3(2.7)	4(3.2)
L3–4	25(10.4)	13(11.3)	12(9.6)
L4–5	93(38.7)	40(34.7)	53(42.4)
** L5–S1**	**115(47.9)**	**59(51.3)**	**56(44.8)**
Prior spine surgery	50(20.8)	27(23.5)	23(18.4)
History of pain			
None to 6wks	8(3.3)	3(2.7)	5(4.0)
6wks to 6mos	97(40.4)	41(35.6)	56(44.8)
** 6mos to 1 yr**	**135(56.3)**	**71(61.7)**	**64(51.2)**
Analgesic drug use			
** Daily**	**180(75.0)**	**92(80.0)**	**88(70.4)**
Weekly	37(15.4)	13(11.3)	24(19.2)
Not regularly	23(9.6)	10(8.7)	13(10.4)
Education			
Elementary	6(2.5)	4(3.5)	5(4.0)
High-school	110(45.8)	55(47.8)	55(44.0)
** Higher**	**114(47.5)**	**51(44.3)**	**63(50.4)**
Post-doctoral	10(4.2)	5(4.4)	2(1.6)
Employment type			
** Employed**	**138(57.5)**	**64(55.7)**	**74(59.2)**
Self-employed	40(16.6)	16(14.0)	24(19.2)
Retired	29(12.1)	14(12.2)	15(12.0)
House worker	10(4.2)	9(7.8)	1(0.8)
Unfit	8(3.3)	5(4.3)	3(2.4)
Unemployed	13(5.4)	6(5.2)	7(5.6)
Student	2(0.1)	1(0.8)	1(0.8)
EQ5D Anxiety & Depression			
** 1**	**137(57.1)**	**57(49.6)**	**80(64.0)**
2	88(36.7)	48(41.7)	40(32.0)
3	15(6.3)	10(8.7)	5(4.0)
5R-STS Time (seconds)	13.04 ± 6.10	12.68 ± 5.20	13.38 ± 6.84

Continuous variables are presented as mean ± SD and categorical variables as frequency (%)

Boldface was used to highlight the most common characterteristic

### Factors associated with the 5R-STS performance

The results of the univariable analysis can be found in Table 2.

**Table 2:** Univariable linear regression analysis of predictive factors for the 5R-STS test time in 240 patients with lumbar degenerative disease

Variable		Univariate analysis	
	RC	95% CI	*p* value
Age	− 0.05	− 0.10 to 0.01	0.117
Gender			
Female	− 0.70	− 2.24 to 0.85	0.375
** Male**		**Reference**	
BMI (kg/m^2^)	− 0.02	− 0.26 to 0.22	0.885
Height (cm)	0.09	0.02 to 0.17	0.019
Weight (kg)	0.05	− 0.01 to 0.11	0.136
Smoking status			
Active smoker	3.28	1.46 to 5.10	< 0.001
Ceased smoking	0.94	− 0.89 to 2.76	0.317
** Never smoked**		**Reference**	
Ability to work			
Full	− 3.50	− 5.38 to 1.62	< 0.001
Limited	− 2.56	− 4.40 to -0.72	0.007
** Unable**		**Reference**	
Indication			
** Disc herniation**		**Reference**	
Stenosis	− 1.71	− 3.75 to 0.32	0.100
DDD	2.97	− 0.43 to 6.37	0.088
Spondylolisthesis	− 2.89	− 6.57 to 0.78	0.124
Index level			
L2–3	0.12	− 4.51 to 4.76	0.957
L3–4	− 3.02	− 5.65 to -0.39	0.025
L4–5	− 0.47	− 2.13 to 1.19	0.580
** L5–S1**		**Reference**	
Prior spine surgery	1.65	− 0.24 to 3.54	0.088
History of pain			
None to 6wks	− 0.19	− 4.52 to 4.14	0.932
6wks to 6mos	1.68	0.10 to 3.26	0.039
** 6mos to 1 yr**		**Reference**	
Analgesic drug use			
** Daily**		**Reference**	
Weekly	− 0.14	− 2.77 to 2.50	0.918
Not regularly	− 2.31	− 4.45 to 0.16	0.036
Education			
Elementary	− 3.10	− 7.86 to 2.16	0.266
High-school	0.91	− 0.69 to 2.51	0.266
** Higher**		**Reference**	
Post-doctoral	− 0.73	− 4.67 to 3.21	0.716
Employment type			
** Employed**		**Reference**	
Self-employed	− 0.46	− 2.59 to 1.67	0.675
Retired	− 2.15	− 4.57 to 0.28	0.085
House worker	− 0.37	− 4.27 to 3.52	0.851
Unfit	4.79	0.46 to 9.12	0.031
Unemployed	0.13	− 3.31 to 3.58	0.940
Student	2.18	− 6.29 to 10.64	0.615
EQ5D Anxiety & Depression			
** 1**		**Reference**	
2	0.53	− 1.09 to 2.15	0.523
3	4.07	0.85 to 7.30	0.014

*BMI*, body mass index; *RC*, regression coefficient; *CI*, confidence interval

Boldface was used to highlight the characteristic to which the RC is referenced too

**Fig. 1:**
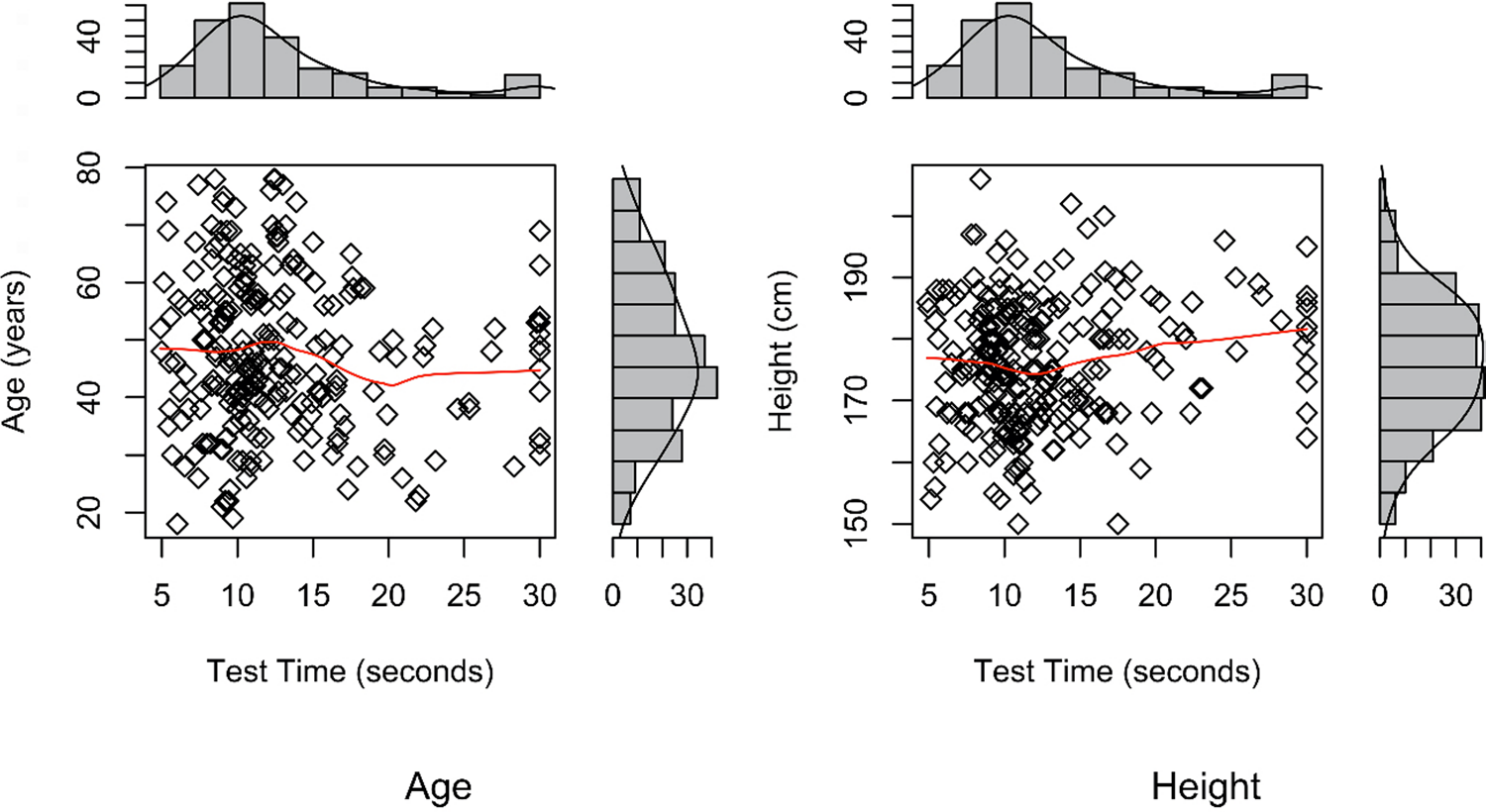
Scatter plots with marginal histograms demonstrating continuous factors associated with 5R-STS test time in 240 adult patients with lumbar degenerative disease using Spearman’s rank correlation

**Fig. 2:**
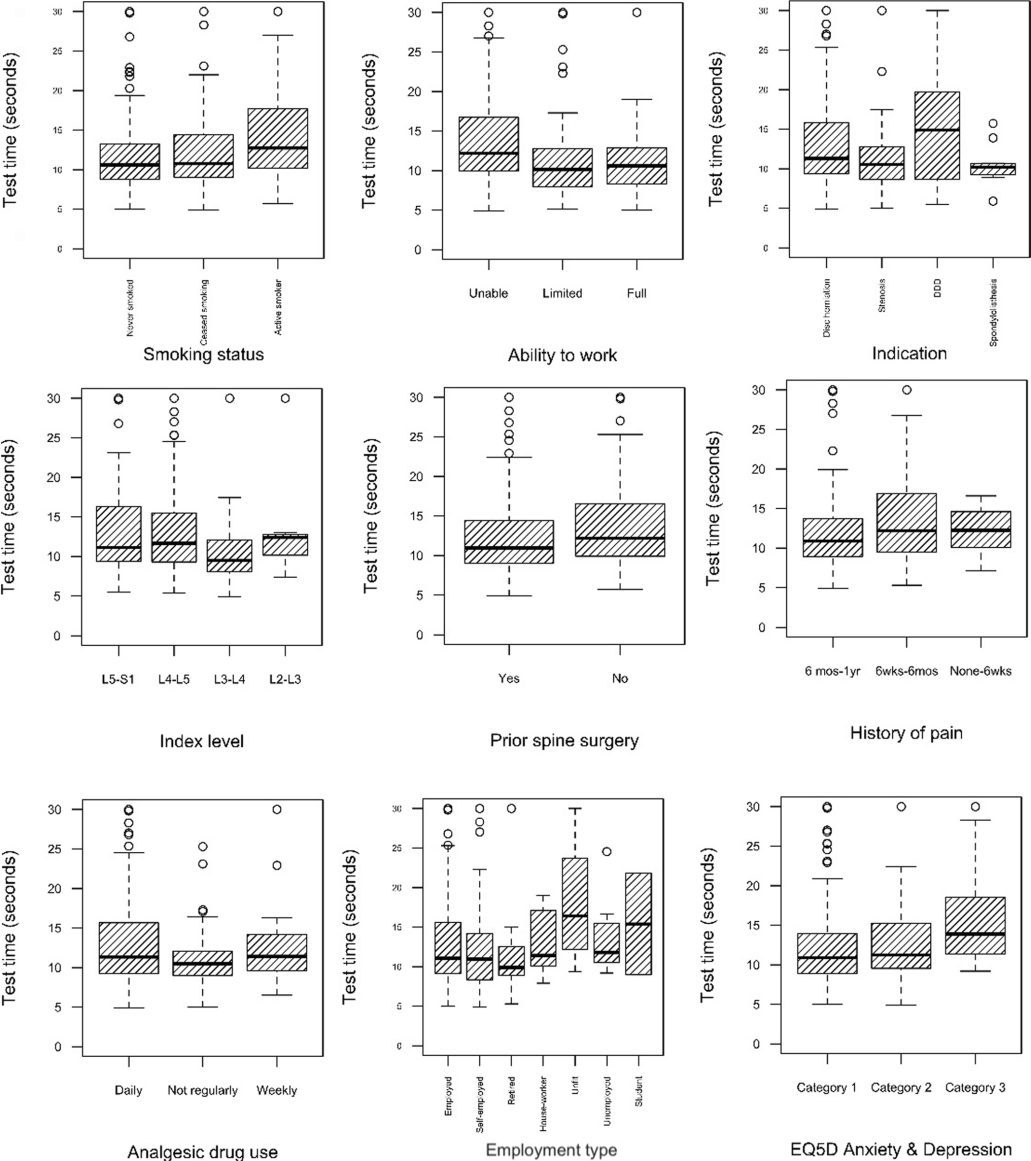
Boxplots of categorical factors associated with 5R-STS test time in 240 adult patients with lumbar degenerative disease

In the final multivariable model including confounders (Table 3), increased height (RC, 0.08; 95%CI, 0.003/0.16; *p* = 0.042) and being an active smoker (RC, 2.44; 95%CI, 0.54/4.33; *p* = 0.012) were significantly associated with increased 5R-STS time, which represented worse performance (Fig. 1). The ability to work fully was significantly associated with decreased 5R-STS that is better 5R-STS performance (RC, − 2.39; 95%CI − 4.39/ − 0.39; *p* = 0.020) (Fig. 2).

**Table 3:** Multivariable linear regression analysis of predictive factors for the 5R-STS test time in patients with lumbar degenerative disease. Variables for inclusion in this final model were selected according to the purposeful variable selection algorithm

Variable		Multivariate analysis	
	RC	95% CI	*p* value
Age	0.02	− 0.05 to 0.10	0.570
Height (cm)	0.08	0.003 to 0.16	0.042*
Smoking status			
Active smoker	2.44	0.56 to 4.32	0.012*
Ceased smoking	0.85	− 1.06 to 2.75	0.383
** Never smoked**		**Reference**	
Ability to work			
Full	− 2.39	− 4.39 to − 0.39	0.020*
Limited	− 1.85	− 3.84 to 0.14	0.070
** Unable**		**Reference**	
Indication			
** Disc herniation**		**Reference**	
Stenosis	− 0.36	− 2.80 to 2.07	0.771
DDD	2.71	− 0.80 to 6.22	0.131
Spondylolisthesis	− 2.72	− 6.39 to 0.95	0.148
Index level			
L2–3	1.07	− 3.85 to 6.00	0.670
L3–4	− 2.12	− 5.06 to 0.83	0.160
L4–5	− 0.13	− 1.83 to 1.57	0.884
** L5–S1**		**Reference**	
Prior spine surgery	0.49	− 1.47 to 2.45	0.624
History of pain			
None to 6wks	− 2.10	− 6.47 to 2.28	0.349
6wks to 6mos	1.33	− 0.33 to 2.99	0.117
** 6mos to 1 yr**		**Reference**	
Analgesic drug us			
** Daily**		**Reference**	
Weekly	0.33	− 2.31 to 2.97	0.806
Not regularly	− 1.70	− 3.90 to 0.50	0.131
Employment type			
** Employed**		**Reference**	
Self-employed	0.06	− 2.12 to 2.24	0.956
Retired	− 0.55	− 3.67 to 2.57	0.729
House	− 0.002	− 3.89 to 3.88	0.999
Unfit	3.80	− 0.56 to 8.16	0.089
Unemployed	− 1.53	− 4.92 to 1.86	0.376
Student	3.04	− 5.23 to 11.31	0.472
EQ5D Anxiety & Depression			
** 1**		**Reference**	
2	0.33	− 1.32 to 1.98	0.699
3	2.69	− 0.69 to 6.07	0.120

*BMI*, body mass index; *RC*, regression coefficient; *CI*, confidence interval

Boldface was used to highlight the characteristic to which the RC is referenced too

Age, surgical indication, index level of pathology, history of previous spine surgery, history of pain, analgesic drug use, employment type, and severity of anxiety and depression symptoms represented by the EQ5D Anxiety& Depression domain did not significantly influence the 5R-STS time but were included in the model due to identified confounding effect.

Gender was not significantly associated with 5R-STS even at univariable analysis (RC, − 0.70; 95%CI, − 2.24/0.85; *p* = 0.375).

## Discussion

The aim of this study was to identify prognostic factors of the 5R-STS test in adult patients with lumbar degenerative disease. There was a positive correlation between height as well as an active smoker status and worse 5R-STS performance. Ability to work fully was associated with better 5R-STS performance. Age, surgical indication, index level of pathology, history of previous spine surgery, history of pain, analgesic drug use, employment type, and severity of anxiety and depression symptoms were not significant influencers of the test but were included in the final model due to confounding effects. Gender did not demonstrate a meaningful influence on the test performance.

Similarly to a population of healthy adult individuals, increased height of patients with LDD correlated with worse 5R-STS performance despite the standardized seat of 43 cm height [14]. This agrees with a study on stroke survivors, which showed that seat height lower than knee height increased the 5R-STS [18]. Therefore, the height of a patient with LDD must be taken into account when interpreting the 5R-STS time and ideally the patient should be sat at knee height level, or height-adjusted test times should be calculated [18, 19].

Multiple studies demonstrated a significant positive correlation between age and 5R-STS performance in adult individuals; however, the participants were often much older than patients from our cohort or categorized into either 20–29 or 80–85 age groups, missing the 48-year mean age of our participants [3, 6, 15]. A more recent study, however, with a mean age of 39 years also identified a significant age-associated increase in 5R-STS in healthy adult individuals which is more suitable for comparison [14]. In our study of patients with LDD, age was not a significant prognostic factor for 5R-STS performance. A possible explanation for this might be that the greatest incidence of LDD falls between 40 and 70 years of age limiting opportunities for identifying a significant correlation [4]. Interestingly, Gautschi et al. [8] found that unadjusted raw TUG time increased with age in their study of patients with LDH and lumbar stenosis, demonstrating the differences between the various tests for OFI.

It has been previously suggested that increased BMI, increased age, and being female are a significant positive prognostic factor for 5R-STS performance in healthy individuals, which is also in agreement with proven risk factors for development of LDD [14, 24, 31]. Contrary to expectations, no such correlation was identified for patients with LDD. It may be theorized that once a symptomatic LDD pathology requiring surgical intervention develops, variation in 5R-STS time is no longer significantly correlated with basic demographic characteristics in contrast to spine-healthy individuals but predominantly influenced by the pathology [14].

Interestingly, in this study, mood-related symptoms were not significantly correlated with 5R-STS time measured by EQ5D Depression & Anxiety domain, which is a validated tool for assessment psychological status [17, 20]. Around 40% of patients with LDD reported a presence of depression and anxiety symptoms, yet the 5R-STS was not influenced by them, emphasizing its objectivity.

The third significant predictor of 5R-STS in patients with LDD was full ability to work. The full ability to work encompasses a range of factors including the physical ability to actually work (degree of OFI), level of experienced pain, pain tolerance threshold, and mental health. In our study, 75% of patients reported using analgesia daily, and nearly 60% experienced spine-associated pain for over half a year. Peters et al. [21] suggests that individuals that experience pain for more than 6 months can acquire a higher pain threshold. If our patient cohort followed that theory, the greater majority would have been able to work. This links to a previous study, which found increased degree of OFI measured by 5R-STS in patients with LDD and high amounts of back pain but not leg pain [13]. So far, the significance of this finding is unclear, but it may be that within a subgroup of patients with LDD, there are patients with a painless motor component. Most of our cohort suffered from LDH. Currently, the most commonly reported symptoms are radiculopathy, sensory abnormalities, and weakness along the distribution of one or more lumbosacral nerves [1]. In two studies of patients undergoing microdiscectomy for LDH, the presence of severe motor deficits was associated with delayed surgical recovery at more than 2 months [30, 33]. Identifying a painless motor deficit in LDD using a simple test holds great potential for improved clinical assessment, especially if it influences postoperative outcomes.

A positive correlation was identified between being an active smoker and worse 5R-STS performance in patients with LDD. However, no relationship between smoking status and TUG time was demonstrated in another study of patients with LDD [11]. The authors, however, did not differentiate between active smokers and ex-smokers, which is a crucial discrepancy given persistent body changes, even years after smoking cessation [2, 11].

The purposeful variable selection algorithm described by Bursac et al. [5] was utilized to identify significant prognostic factors of 5R-STS in patients with LDD. This allowed us to also identify which factors may not be significant predictors but may still indirectly influence 5R-STS performance through confounding. Accordingly, variables with confounding effect — such as age, height, surgical indication, index level of pathology, history of previous spine surgery, history of pain, analgesic drug use, employment type, and severity of anxiety and depression symptoms — were recognized and ought to be accounted for in future studies of patients with LDD where 5R-STS is used.

### Limitations

One of the first limitations is the uneven distribution of patients among certain subcategories, more specifically indication. Therefore, the results of this study should not be applied to patients with individual spinal pathology but rather provide an overview of prognostic factors for a range of LDD, because of a lack of statistical power for these subgroup analyses. Since LDH was the most predominant indication in this patient cohort — contributing 72.5% of all patients — the results certainly are powerful enough for this specific patient cohort. Further research to identify prognostic factors of 5R-STS time in individual LDD conditions is encouraged.

The presence of other chronic conditions in this study was not clearly reported — this may unknowingly have influenced the 5R-STS performance. However, our exclusion of patients with hip and/or knee prosthetics and walking aids meant that individuals with comorbidities severely affecting their mobility were not included. This is supported by a weak and inconsistent association between presence of medical comorbidities and degree of OFI measured by another objective test, TUG, in patients with LDD [26].

In our study, there is no differentiation between physically active and predominantly stationary employment in relation to the “working ability” category. It would seem that patients with LDD and heavy lifting-focused jobs should be more limited in their working ability than, e.g., office workers [16]. However, individuals with sedentary occupations may also be limited by LDD symptoms due to related long-term axial loading and increased disc pressure [1, 16, 32].

Due to the incidence of LDD in the middle-aged group, another limitation that is challenging to overcome is not fully being able to identify prognostic factors of 5R-STS time in patients with LDD across other individual age groups.

Lastly, patients in this cohort were from a Dutch specialized short-stay clinic and had a diagnosis of LDD eligible for surgical intervention. Therefore, the identified prognostic factors in this study should be applied to patients with advanced LDD. Studies from other geographical areas are encouraged.

## Conclusions

Greater height, being an active smoker, and inability to work are significant prognostic factors of worse 5R-STS performance in patients with LDD. This requires that 5R-STS test time thresholds for OFI are adjusted for these factors. Age, surgical indication, index level of pathology, history of previous spine surgery, history of pain, analgesic drug use, employment type, and severity of anxiety and depression symptoms represent important confounders of 5R-STS performance and should thus be included in future studies utilizing the 5R-STS as an outcome measure.

## Supplementary information

Below is the link to the electronic supplementary material.

Supplementary file1 (DOCX 25 KB)
