# Supplementary material for: Predictors of five-repetition sit-to-stand test performance in patients with lumbar degenerative disease
Source: Acta Neurochir (Wien). 2022 Dec 7;165(1):107–15. doi: 10.1007/s00701-022-05441-1 (PMC9840589; doi:10.1007/s00701-022-05441-1)
Supplement: Supplementary file 1 — Supplementary file1 (DOCX 25 KB) [file 701_2022_5441_MOESM1_ESM.docx]

**Supplementary Content 1** Statistical Code. R Code for the statistical analysis figure rendering. The code was executed in R Version 3.5.2 (The R Foundation for Statistical Computing, Vienna, Austria) on a machine running macOS Catalina Version 10.15.7. The raw data will be made available by the authors on request.

#R Script - 5R-STS - Predictors of performance

#Import data patient

library(readxl)

df <- read_excel("Desktop/victor/5sitandstandtest/prognosticfactors/datapredictorspt.xlsx")

View(df)

remove(df)

remove(df)

remove(fitmulti)

remove(fituni)

remove(X5RSTStesthome)

#Codinglegend

Female=0, Male=1;

Higher=1 High-school=2, Post-doctoral=3, Elementary=4

Employed=1, student=2, self-employed=3,retired=4, house (home-maker)=5, unemployed=6

Never smoked=1, ex smoker=2, smoking=3

#Reformat variables

df$indication <- factor(df$indication)

df$level <- factor(df$level)

df$historyofpain <- factor(df$historyofpain)

df$previoussurgery <- factor(df$previoussurgery)

df$gender <- factor(df$gender)

df$education <- factor(df$education)

df$worksituation <- factor(df$worksituation)

df$analgesia <- factor(df$analgesia)

df$smoking <- factor(df$smoking)

df$workability <- factor(df$workability)

df$mood <- factor(df$mood)

df$testtime <- as.numeric(df$testtime)

df$age <- as.numeric(df$age)

df$height <- as.numeric(df$height)

df$weight <- as.numeric(df$weight)

df$BMI <- as.numeric(df$BMI)

df$EQ5DINDEX <- as.numeric(df$EQ5DINDEX)

df$EQ5DVAS <- as.numeric(df$EQ5DVAS)

t.test(df$testtime ~ df$mood, paired = F)

#Group Comparison

mean(df$testtime)

sd(df$testtime)

#Mean per Group

mean(df$testtime[df$gender==1 & df$age>50 & df$age<61], na.rm = T)

sd(df$testtime[df$gender==1 & df$age>20 & df$age<31], na.rm = T)

quantile(df$testtime[df$gender==1 & df$age>50 & df$age<61], na.rm = T, probs = 0.99)

#Calculating reference values

a <- (df$testtime[df$gender==0 & df$age>20 & df$age<31], na.rm = T)

mean(a)

sd(a)

quantile(a, probs = 0.99)

#Calculating range of variable

range(df$height[df$gender==0], na.rm = T)

table(df$indication)

table(df$smoking)

table(df$height, df$gender)

table(df$gender)

table(df$age>20)

#Univariate linear regression

fituni <- glm(testtime ~ age, df, family = "gaussian")

summary(fituni)

confint(fituni)

fituni <- glm(testtime ~ BMI, df, family = "gaussian")

summary(fituni)

confint(fituni)

fituni <- glm(testtime ~ weight, df, family = "gaussian")

summary(fituni)

confint(fituni)

fituni <- glm(testtime ~ indication, df, family = "gaussian")

summary(fituni)

confint(fituni)

fituni <- glm(testtime ~ level, df, family = "gaussian")

summary(fituni)

confint(fituni)

fituni <- glm(testtime ~ historyofpain, df, family = "gaussian")

summary(fituni)

confint(fituni)

fituni <- glm(testtime ~ previoussurgery, df, family = "gaussian")

summary(fituni)

confint(fituni)

fituni <- glm(testtime ~ gender, df, family = "gaussian")

summary(fituni)

confint(fituni)

fituni <- glm(testtime ~ education, df, family = "gaussian")

summary(fituni)

confint(fituni)

fituni <- glm(testtime ~ worksituation, df, family = "gaussian")

summary(fituni)

confint(fituni)

fituni <- glm(testtime ~ analgesia, df, family = "gaussian")

summary(fituni)

confint(fituni)

fituni <- glm(testtime ~ smoking, df, family = "gaussian")

summary(fituni)

confint(fituni)

fituni <- glm(testtime ~ workability, df, family = "gaussian")

summary(fituni)

confint(fituni)

fituni <- glm(testtime ~ mood, df, family = "gaussian")

summary(fituni)

confint(fituni)

#Multivariate linear regression

fitmulti <- glm(testtime ~ age + height + weight + smoking + workability + indication + level + previoussurgery + historyofpain + analgesia + worksituation + mood, df, family = "gaussian")

summary(fitmulti)

confint(fitmulti)

fitmulti <- glm(testtime ~ age + BMI + weight + worksituation + mood, df, family = "gaussian")

summary(fitmulti)

confint(fitmulti)

#Assessing for confounding

fitmulti <- glm(testtime ~ age + height + weight + smoking + workability + indication + level + previoussurgery + historyofpain + analgesia + worksituation, df, family = "gaussian")

summary(fitmulti)

confint(fitmulti)

#Final model for patients

fitmulti <- glm(testtime ~ age + height + smoking + workability + indication + level + previoussurgery + historyofpain + analgesia + worksituation + mood, df, family = "gaussian")

summary(fitmulti)

confint(fitmulti)

########################################################################################################################################################################################

#Correlation Plots

library(psych)

jpeg(filename = "age.jpeg", width = 3.5, height = 4.25, units = "in", res = 2000)

scatterHist(df$testtime, df$age, ellipse = F, method = "spearman", col = 1, pch = 4, xlab = "Test Time (seconds)", ylab = "Age (years)", title = "")

mtext("Age", side=1, line=4)

dev.off()

cor.plot(df)

p <- density(df$testtime)

plot(p)

#Boxplots

#Boxplot (Cont.)

jpeg(filename = "analgesia.jpeg", width = 3.5, height = 4.25, units = "in", res = 2000)

b.x <- boxplot(ylab = "Test time (seconds)", df$testtime ~ df$analgesia , density = 20, angle = 45, col = "LIGHTgray", horiz = F, names = c("Daily","Not regularly","Weekly"), xlab = "", las = 1 , ylim = c(0,30))

b.x <- shaded.bxp(b.x, density = 20, boxfill = 1, ylab = "Test time (seconds)", names = c("Daily","Not regularly","Weekly"), cex.axis = 0.7, xlab = "", las = 1 , ylim = c(0,30))

mtext("Analgesic drug use", side=1, line=4)

dev.off()

#Run this Code FIRST to obtain shaded boxplots:

shaded.bxp <- function (z, notch = FALSE, width = NULL, varwidth = FALSE, outline = TRUE,

notch.frac = 0.5, log = "", border = par("fg"), pars = NULL,

frame.plot = axes, horizontal = FALSE, add = FALSE, at = NULL,

show.names = NULL, density=NULL, angle=45, ...)

{

pars <- c(list(...), pars)

pars <- pars[unique(names(pars))]

bplt <- function(x, wid, stats, out, conf, notch, xlog, i, density, angle=45, boxfill) {

ok <- TRUE

if (!any(is.na(stats))) {

xP <- if (xlog)

function(x, w) x * exp(w)

else function(x, w) x + w

wid <- wid/2

if (notch) {

ok <- stats[2L] <= conf[1L] && conf[2L] <= stats[4L]

xx <- xP(x, wid * c(-1, 1, 1, notch.frac, 1,

1, -1, -1, -notch.frac, -1))

yy <- c(stats[c(2, 2)], conf[1L], stats[3L],

conf[2L], stats[c(4, 4)], conf[2L], stats[3L],

conf[1L])

}

else {

xx <- xP(x, wid * c(-1, 1, 1, -1))

yy <- stats[c(2, 2, 4, 4)]

}

if (!notch)

notch.frac <- 1

wntch <- notch.frac * wid

xypolygon(xx, yy, lty = "blank", col = boxfill[i], density=density[i], angle=angle[i])

xysegments(xP(x, -wntch), stats[3L], xP(x, +wntch),

stats[3L], lty = medlty[i], lwd = medlwd[i],

col = medcol[i], lend = 1)

xypoints(x, stats[3L], pch = medpch[i], cex = medcex[i],

col = medcol[i], bg = medbg[i])

xysegments(rep.int(x, 2), stats[c(1, 5)], rep.int(x,

2), stats[c(2, 4)], lty = whisklty[i], lwd = whisklwd[i],

col = whiskcol[i])

xysegments(rep.int(xP(x, -wid * staplewex[i]), 2),

stats[c(1, 5)], rep.int(xP(x, +wid * staplewex[i]),

2), stats[c(1, 5)], lty = staplelty[i], lwd = staplelwd[i],

col = staplecol[i])

xypolygon(xx, yy, lty = boxlty[i], lwd = boxlwd[i],

border = boxcol[i], density=density[i], angle=angle[i], col=boxfill[i])

if ((nout <- length(out))) {

xysegments(rep(x - wid * outwex, nout), out,

rep(x + wid * outwex, nout), out, lty = outlty[i],

lwd = outlwd[i], col = outcol[i])

xypoints(rep.int(x, nout), out, pch = outpch[i],

lwd = outlwd[i], cex = outcex[i], col = outcol[i],

bg = outbg[i])

}

if (any(inf <- !is.finite(out))) {

warning(sprintf(ngettext(length(unique(out[inf])),

"Outlier (%s) in boxplot %d is not drawn",

"Outliers (%s) in boxplot %d are not drawn"),

paste(unique(out[inf]), collapse = ", "), x),

domain = NA)

}

}

return(ok)

}

if (!is.list(z) || 0L == (n <- length(z$n)))

stop("invalid first argument")

if (is.null(at))

at <- 1L:n

else if (length(at) != n)

stop("'at' must have same length as 'z$n', i.e. ", n)

if (is.null(z$out))

z$out <- numeric()

if (is.null(z$group) || !outline)

z$group <- integer()

if (is.null(pars$ylim))

ylim <- range(z$stats[is.finite(z$stats)], if (outline) z$out[is.finite(z$out)],

if (notch) z$conf[is.finite(z$conf)])

else {

ylim <- pars$ylim

pars$ylim <- NULL

}

if (is.null(pars$xlim))

xlim <- c(0.5, n + 0.5)

else {

xlim <- pars$xlim

pars$xlim <- NULL

}

if (length(border) == 0L)

border <- par("fg")

dev.hold()

on.exit(dev.flush())

if (!add) {

plot.new()

if (horizontal)

plot.window(ylim = xlim, xlim = ylim, log = log,

xaxs = pars$yaxs)

else plot.window(xlim = xlim, ylim = ylim, log = log,

yaxs = pars$yaxs)

}

xlog <- (par("ylog") && horizontal) || (par("xlog") && !horizontal)

pcycle <- function(p, def1, def2 = NULL) rep(if (length(p)) p else if (length(def1)) def1 else def2,

length.out = n)

p <- function(sym) pars[[sym, exact = TRUE]]

boxlty <- pcycle(pars$boxlty, p("lty"), par("lty"))

boxlwd <- pcycle(pars$boxlwd, p("lwd"), par("lwd"))

boxcol <- pcycle(pars$boxcol, border)

boxfill <- pcycle(pars$boxfill, par("bg"))

density <- rep(density, length.out=n)

density <- rep(density, length.out=n)

angle <- rep(angle, length.out=n)

boxwex <- pcycle(pars$boxwex, 0.8 * {

if (n <= 1)

1

else stats::quantile(diff(sort(if (xlog)

log(at)

else at)), 0.1)

})

medlty <- pcycle(pars$medlty, p("lty"), par("lty"))

medlwd <- pcycle(pars$medlwd, 3 * p("lwd"), 3 * par("lwd"))

medpch <- pcycle(pars$medpch, NA_integer_)

medcex <- pcycle(pars$medcex, p("cex"), par("cex"))

medcol <- pcycle(pars$medcol, border)

medbg <- pcycle(pars$medbg, p("bg"), par("bg"))

whisklty <- pcycle(pars$whisklty, p("lty"), "dashed")

whisklwd <- pcycle(pars$whisklwd, p("lwd"), par("lwd"))

whiskcol <- pcycle(pars$whiskcol, border)

staplelty <- pcycle(pars$staplelty, p("lty"), par("lty"))

staplelwd <- pcycle(pars$staplelwd, p("lwd"), par("lwd"))

staplecol <- pcycle(pars$staplecol, border)

staplewex <- pcycle(pars$staplewex, 0.5)

outlty <- pcycle(pars$outlty, "blank")

outlwd <- pcycle(pars$outlwd, p("lwd"), par("lwd"))

outpch <- pcycle(pars$outpch, p("pch"), par("pch"))

outcex <- pcycle(pars$outcex, p("cex"), par("cex"))

outcol <- pcycle(pars$outcol, border)

outbg <- pcycle(pars$outbg, p("bg"), par("bg"))

outwex <- pcycle(pars$outwex, 0.5)

width <- if (!is.null(width)) {

if (length(width) != n | any(is.na(width)) | any(width <=

0))

stop("invalid boxplot widths")

boxwex * width/max(width)

}

else if (varwidth)

boxwex * sqrt(z$n/max(z$n))

else if (n == 1)

0.5 * boxwex

else rep.int(boxwex, n)

if (horizontal) {

xypoints <- function(x, y, ...) points(y, x, ...)

xypolygon <- function(x, y, ...) polygon(y, x, ...)

xysegments <- function(x0, y0, x1, y1, ...) segments(y0,

x0, y1, x1, ...)

}

else {

xypoints <- points

xypolygon <- polygon

xysegments <- segments

}

ok <- TRUE

for (i in 1L:n) ok <- ok & bplt(at[i], wid = width[i], stats = z$stats[,

i], out = z$out[z$group == i], conf = z$conf[, i], notch = notch,

xlog = xlog, i = i, density=density, angle=angle, boxfill=boxfill)

if (!ok)

warning("some notches went outside hinges ('box'): maybe set notch=FALSE")

axes <- is.null(pars$axes)

if (!axes) {

axes <- pars$axes

pars$axes <- NULL

}

if (axes) {

ax.pars <- pars[names(pars) %in% c("xaxt", "yaxt", "xaxp",

"yaxp", "las", "cex.axis", "col.axis", "format")]

if (is.null(show.names))

show.names <- n > 1

if (show.names)

do.call("axis", c(list(side = 1 + horizontal, at = at,

labels = z$names), ax.pars))

do.call("Axis", c(list(x = z$stats, side = 2 - horizontal),

ax.pars))

}

do.call("title", pars[names(pars) %in% c("main", "cex.main",

"col.main", "sub", "cex.sub", "col.sub", "xlab", "ylab",

"cex.lab", "col.lab")])

if (frame.plot)

box()

invisible(at)

}
